# Supplementary material for: Content-rich biological network constructed by mining PubMed abstracts
Source: BMC Bioinformatics. 2004 Oct 8;5:147. doi: 10.1186/1471-2105-5-147 (PMC528731; doi:10.1186/1471-2105-5-147)
Supplement: Additional File 5 — The original Chilibot query results of the term "long-term potentiation (LTP)" and 22 other terms, limiting the latest references analyzed to the years 1990, 1995, 2000, and 2004. [file 1471-2105-5-147-S5.bz2 › chilibotAdditionalFile5/ltp2004/html/NMDA_SYNAPTOPHYSIN.html]

 


 **NMDA** and **SYNAPTOPHYSIN** 
  
Found 43 abstracts in PubMed,  **30 abstracts were retrieved and analyzed**.  


---

 Search Google  |
 PDF files only 
|  EDU domain only 

---

**Interactive relationship** (e.g. stimulation, inhibition, etc)

- Consistently, immunostaining indicated significant increases in the proportion of the clusters of NR1, an  **NMDA**  receptor subunit, which were associated with the accumulation of  **synaptophysin** , a presynaptic marker, in cultured E18 neurons between 7 and 14 DIV.  Ref: 11835315 J Neurosci Res, 2002
- ErbB 4 colocalizes with PSD 95 and  **NMDA**  receptors at a subset of excitatory synapses apposed to  **synaptophysin**  positive presynaptic terminals.  Ref: 10725395 Proc Natl Acad Sci U S A, 2000
- Fourth, many PSD 95 immunopositive puncta were apposed closely to  **synaptophysin**  positive nerve terminals and overlapped with  **NMDA**  receptor subunits.  Ref: 11027400 J Comp Neurol, 2000
- The formation of functional  **NMDA**  receptors also coincided with the appearance of synapsin I and  **synaptophysin** .  Ref: 11920728 J Neurobiol, 2002

**Parallel relationship** (e.g. studied together, co-existance, homology, etc.)

- Semi quantitative Western blot techniques were applied with the use of antibodies that recognize either the epsilon1 NR2A, epsilon2 NR2B or zeta1 NR1 subunits of the  **NMDA**  receptor or a synaptic terminal protein,  **synaptophysin** .  Ref: 11869807 Brain Res Mol Brain Res, 2002
- Participation of  **NMDA**  receptors in reduced magnesium induced synaptic events was supported by the localization of the NR1 subunit of the  **NMDA**  receptor with the presynaptic vesicular protein  **synaptophysin** .  Ref: 14534286 J NeurophysiolJ Neurophysiol, 2002
- Studies of gene transcription have provided replicable evidence of decreased expression of mRNAs for  **synaptophysin** , GAP 43, cholecystokinin, and non  **NMDA**  glutamate receptor subunits, particularly in CA 3 4.  Ref: 10071707 Biol Psychiatry, 1999
- One of the retinal cell clones, designated RGC 5, was positive for Thy 1, Brn 3C, Neuritin,  **NMDA**  receptor, GABA B receptor, and  **synaptophysin**  expression and negative for GFAP, HPC 1, and 8A1, suggesting that it represented a putative RGC clone.  Ref: 11165366 Brain Res Mol Brain Res, 2001
- Using these antibodies, in conjunction with GluR2 specific and  **synaptophysin**  antibodies, we have directly localized and quantified surface expressed native AMPA and  **NMDA**  receptors on cultured living hippocampal neurons during development.  Ref: 11050112 J Neurosci, 2000
